# Rosiglitazone Suppresses *In Vitro* Seizures in Hippocampal Slice by Inhibiting Presynaptic Glutamate Release in a Model of Temporal Lobe Epilepsy

**DOI:** 10.1371/journal.pone.0144806

**Published:** 2015-12-14

**Authors:** Shi-Bing Wong, Sin-Jhong Cheng, Wei-Chen Hung, Wang-Tso Lee, Ming-Yuan Min

**Affiliations:** 1 Department of Pediatrics, Taipei Tzu Chi General Hospital, Buddhist Tzu Chi Medical Foundation, Taipei, Taiwan; 2 Institute of Zoology, College of Life Science, National Taiwan University, Taipei, Taiwan; 3 Neuroscience Program in Academia Sinica, Taipei, Taiwan; 4 Institute of Biomedical Sciences; Academia Sinica, Taipei, Taiwan; 5 Department of Pediatrics, National Taiwan University Hospital, Taipei, Taiwan; University of Exeter, UNITED KINGDOM

## Abstract

Peroxisomal proliferator-activated receptor gamma (PPARγ) is a nuclear hormone receptor whose agonist, rosiglitazone has a neuroprotective effect to hippocampal neurons in pilocarpine-induced seizures. Hippocampal slice preparations treated in Mg^2+^ free medium can induce ictal and interictal-like epileptiform discharges, which is regarded as an in vitro model of N-methyl-D-aspartate (NMDA) receptor-mediated temporal lobe epilepsy (TLE). We applied rosiglitazone in hippocampal slices treated in Mg^2+^ free medium. The effects of rosiglitazone on hippocampal CA1-Schaffer collateral synaptic transmission were tested. We also examined the neuroprotective effect of rosiglitazone toward NMDA excitotoxicity on cultured hippocampal slices. Application of 10μM rosiglitazone significantly suppressed amplitude and frequency of epileptiform discharges in CA1 neurons. Pretreatment with the PPARγ antagonist GW9662 did not block the effect of rosiglitazone on suppressing discharge frequency, but reverse the effect on suppressing discharge amplitude. Application of rosiglitazone suppressed synaptic transmission in the CA1-Schaffer collateral pathway. By miniature excitatory-potential synaptic current (mEPSC) analysis, rosiglitazone significantly suppressed presynaptic neurotransmitter release. This phenomenon can be reversed by pretreating PPARγ antagonist GW9662. Also, rosiglitazone protected cultured hippocampal slices from NMDA-induced excitotoxicity. The protective effect of 10μM rosiglitazone was partially antagonized by concomitant high dose GW9662 treatment, indicating that this effect is partially mediated by PPARγ receptors. In conclusion, rosiglitazone suppressed NMDA receptor-mediated epileptiform discharges by inhibition of presynaptic neurotransmitter release. Rosiglitazone protected hippocampal slice from NMDA excitotoxicity partially by PPARγ activation. We suggest that rosiglitazone could be a potential agent to treat patients with TLE.

## Introduction

Epilepsy is the second most common neurological disorder with a prevalence in developed countries of four to ten cases per 1,000. Partial epilepsies account for about 60% of all adult epilepsy cases, with temporal lobe epilepsy (TLE) being the most common type [1]. More than 60% of patients with focal seizures achieve seizure freedom from anti-epileptic drugs (AED) [2]. However, there are still a large number of patients suffering from recurrent seizures. Several molecular mechanisms have been reported to be related to recurrent seizures, including low brain gamma amino butyric acid (GABA) levels [3] and changes in either glutamate levels or glutamate transporters[4]. High extracellular glutamate has been found in human epileptogenic hippocampus during both inter-ictal periods[5] and complex partial seizures[6]. Therefore, targeting glutamate receptors may be a potential treatment of choice in the future.

A low-magnesium medium can induce ictal and interictal-like epileptiform discharges in hippocampal slice preparations, which is regarded as an in vitro model of TLE [7–9]. Those epileptiform discharges are mediated by the N-methyl-D-aspartate (NMDA) receptor [10] and can be blocked by the NMDA-antagonist 3,3(2-carboxy-piperazine-4-yl)propyl-1-phosphonate (CPP) [8]. Thus, this model can be used as a platform to study the pathogenesis and treatment of TLE. However, the use of broad-spectrum NMDA receptor antagonists has failed in clinical trials due to serious side effects [11].

Rosiglitazone was released by GlaxoSmithKline in 1999 and belongs to the thiazolidinedione (TZD) class of drugs. The TZD class drugs are potent, exogenous agonists of the peroxisome proliferator-activated receptor gamma (PPARγ)[12]. PPARγ is a nuclear hormone receptor and plays an important role in adipocyte differentiation, lipid biogenesis, glucose homeostasis, and immunomodulation[13]. The PPARγ receptor is also found in the CNS, primarily localized to hippocampal CA 1 pyramidal cells and the granular and polymorphic layers of the dentate gyrus[14]. PPAR ligands have been shown to induce significant neuroprotection in animal models of focal ischemia and spinal cord injury by multiple mechanisms, such as prevention of microglial activation, and inhibition of inflammatory cytokine and chemokine expression [13]. In pilocarpine-induced status epilepticus in rats, rosiglitazone significantly reduced hippocampal neuronal loss by suppression of CD40 and tumor necrosis factor-alpha expression, microglial activation, and reactive oxygen species (ROS) production [15, 16]. These effects were blocked by PPARγ antagonist, suggesting that activation of the PPARγ pathway might provide neuroprotection during status epilepticus.

The severity of pentylenetetrazole induced seizures have been suppressed by pioglitazone (another TZD class ligand), with similar efficacy as valproate [17] suggesting that activation of the PPARγ pathway directly suppresses hyperactive neuronal activity. As rosiglitazone and pioglitazone have been shown to reduce calcium influx in primary hippocampal cultured neurons through voltage-gated Ca^2+^ channels and NMDA receptors, respectively [18], rosiglitazone might have the potential to suppress seizures via direct action on Ca^2+^. To test this hypothesis, we applied rosiglitazone to epileptic hippocampal slices triggered by Mg^2+^-free medium. We also investigated the effects of rosiglitazone toward synaptic transmission at the CA1-Schaffer collateral pathway, and the ability of rosiglitazone to rescue hippocampal slice cultures from NMDA excitotoxicity. We found that rosiglitazone can suppress NMDA receptor-mediated epileptiform discharges by inhibition of presynaptic neurotransmitter release. Rosiglitazone can also protect hippocampal slice from NMDA excitotoxicity partially by PPARγ activation, which had never been reported before.

## Material and Methods

### Animals

The use of animals in this study was approved by the Ethical Committee for Animal Research of the Buddhist Taipei Tzu-Chi General Hospital (101-IACUC-003, 101-IACUC-017) in accordance with National Institutes of Health guidelines. Every effort was made to minimize the number of animals used and their suffering.

### Tissue preparation for electrophysiology experiments

Adult Sprague-Dawley rats (150–250 g) were anesthetized with isoflurane and decapitated. The brains were quickly removed and placed in ice-cold ACSF containing the following (in mM): 119 NaCl, 2.5 KCl, 1.3 MgSO_4_, 26.2 NaHCO_3_, 1 NaH_2_PO_4_, 2.5 CaCl_2_, and 11 glucose, with the pH maintained at 7.4 by gassing with 95% O_2_/5% CO_2_. The hippocampi were transversely sliced into 450 μm sections with a tissue slicer (D.S.K. Super Microslicer Zero 1; Dosaka EM, Kyoto, Japan), and transferred to an interface-type holding chamber at room temperature (25°C). The slices were allowed to recover for at least 90 minutes and then were transferred to an immersion-type recording chamber and perfused at 2–3 ml/min with ACSF at room temperature.

For epileptiform discharge recording, bipolar stainless steel stimulating electrodes (Frederick Haer Company, Bowdoinham, ME) were placed in the stratum pyramidale of the CA1 area. The recordings were performed at room temperature. Hippocampal slices were perfused with Mg^2+^ free ACSF containing the following (in mM): 119 NaCl, 4 KCl, 26.2 NaHCO_3_, 1 NaH_2_PO_4_, 2.5 CaCl2, and 11 glucose. Two kinds of spontaneous events can be recorded under Mg^2+^-free ACSF perfusion. One is a prolonged epileptiform discharge that lasts several seconds and displays both tonic and clonic electrographic components that originate from entorhinal cortex. The other type of spontaneous events have durations shorter than few seconds and are thought to be CA3-driven (for review, see [19]). Because we cut down the entorhinal cortex during slice preparation, there were only few slices present with ictal discharges, which were excluded in this study. The data was collected at room temperature. The spontaneous events recorded in the stratum pyramidale of the CA1 area were counted for spike frequencies and amplitudes manually.

For extracellular field potential recording, a glass pipette filled with 3M NaCl was positioned in the stratum radiatum of the CA1 area, and the field excitatory postsynaptic potential (fEPSP) was amplified by a differential amplifier (DP-301, Warner Instrument, USA) and recorded by PowerLab data acquisition hardware (ADInstrument, USA). Data was collected using Chart software (ADInstrument, USA). Bipolar stainless steel stimulating electrodes (Frederick Haer Company, Bowdoinham, ME) were placed in the stratum radiatum to stimulate Schaffer collateral branches. Stable baseline fEPSP activity was recorded once per minute for at least 15 minutes, with the initial slopes in 1 millisecond width immediately after presynaptic volley measured for data analysis. Synaptic responses were normalized to the average of the baseline. The data was collected at room temperature.

For miniature excitatory-postsynaptic current (mEPSC) recording, the slices were placed in a chamber mounted on an upright microscope (BX51WI, Olympus Optical Co., Ltd, Tokyo,Japan) and perfused with ACSF (osmolarity set to 310 mOsm with sucrose) at 1ml/min at room temperature. The resistances of patch pipettes were 6–10 MΩ when filled with the pipette solutions consisted of (in mM): 131 potassium gluconate, 20 KCl, 8 NaCl, 10 HEPES, 2 EGTA, 2 ATP, and 0.3 GTP (pH 7.2 to 7.3, osmolarity = 300–305 mOsm). All experiments were recording using a Multiclamp 700B amplifier in voltage-clamp mode. Signals were low pass–filtered at 2 kHz and digitized at 10 kHz with a Micro 1401 interface running Spike2 software (Cambridge Electronic Design, Cambridge, UK). Miniature excitatory postsynaptic currents (mEPSCs) were isolated in the presence of 1μM Tetradotoxin (TTX), 1μM strychnine and 100μM picrotoxin, and maintained at a holding potential of −70 mV at room temperature. The mEPSCs were selected manually with MiniAnalysis software (Synaptosoft, NJ, USA). The amplitude and interevent interval were counted by MiniAnalysis software.

### Preparation of hippocampal slice cultures for neurotoxicity experiments

Hippocampal slice cultures (HSC) for neurotoxicity experiments were prepared from 7 to 9-day-old Sprague Dawley rats (n = 18; BioLASCO Taiwan Co., Ltd, Taiwan) using standard methods[20] modified in our lab. In brief, after being anesthetized with isoflurane, the rats were decapitated and the brain was quickly removed and placed in ice-cold ACSF. The hippocampi were then transversely sliced into 350μm sections with a tissue slicer (D.S.K. Super Microslicer Zero 1; Dosaka EM, Kyoto, Japan). The entorhinal cortex was then removed and the middle 4–6 slices of each hippocampus were placed onto tissue culture membrane inserts (Millicell-CM, Millipore, Billerica, MA) within 6-well culture trays with 1 ml of slice culture medium per well. The slice culture medium consisted of 50% minimal essential medium, 25% Hank’s balanced salt solution, 25% heat-inactivated horse serum, 0.5% glucose, 2mM Glutamax, and 2% penicillin–streptomycin. Culture medium was replaced every 2 or 3 days. Seven to eight days later, the HSCs were used in experiments. All media was obtained from Gibco (Life Technologies, Carlsbad, USA).

### Propidium iodide fluorescence measurements

Cell viability was determined using propidium iodide (PI) fluorescence measurement. It is a polar compound that can only enter into dead and dying neurons and binds to nucleic acid resulting in a red fluorescence emission at 630 nm upon excitation at 495 nm with an intensity linearly related to the number of dead cells. PI was applied at 2 μM, 24 hours before fluorescence measurements using an inverted microscope (TE200UA, Nikon, Japan) attached to a digital camera (Evolution QEi, media Cybernetics, USA). Cell death was expressed as a percent increase of mean pixel value of matched controls and recorded using Photoshop software (Adobe, San Jose, USA). All measurements were made after subtracting background fluorescence obtained from a region positioned immediately outside the culture. To assess differential cell death by region, cell regions were identified and circled as regions of interest in the phase contrast image. PI staining was measured densitometrically in each of the 3 cell regions (CA1, CA3, dentate gyrus) using Photoshop software.

### Drugs

The chemicals used for the ACSF were purchased from Merck (Frankfurt, Germany). Rosilgitazone and GW9662 were purchased from Cayman (Ann Harbor, Michigan, USA) and were dissolved in dimethylsulfoxide (DMSO) before experiments. _DL_-2-Amino-5-phosphonopentanoic acid (APV) was purchased from Tocris Cookson (Bristol, UK). Strychnine, NMDA and propidium iodide were purchased from Sigma (St Louis, MO, USA). TTX was purchased from AffixScientific (Fremont, California, USA). Picrotoxin was purchased from abcam (Cambridge, UK). Strychnine, TTX, APV and NMDA were dissolved in distilled water shortly before experiments.

### Data and statistical analysis

All data were presented as the mean±standard error. For statistical analysis with spontaneous epileptiform spikes induced by Mg^2+^ free medium, one-way ANOVA with post-hoc analysis was used to compare the effect of different experimental conditions. To analyze the changes of EPSP slopes, repeated-measured ANOVA was used to compare the changes between baseline and post-treatment. Post-hoc analysis was performed to determine the differences between each treatment groups. For analysis of mEPSC, paired-t test was performed to compare amplitudes and frequencies of mEPSCs before and after treatment. Kolmogorov–Smirnov test (K-S test) was used to compare cumulative probabilities before and after rosiglitazone treatment. To analyze PI density after NMDA exposure between different treatment groups, one way ANOVA with post-hoc analysis was applied. Either Least Significant Difference (LSD) or Games-Howell test were applied for post-hoc analysis. P<0.05 was considered statistically significant.

## Results

### Effect of rosiglitazone 10μM on epileptiform discharges in hippocampal slices induced by Mg^2+^ free ACSF

**Fig 1:**
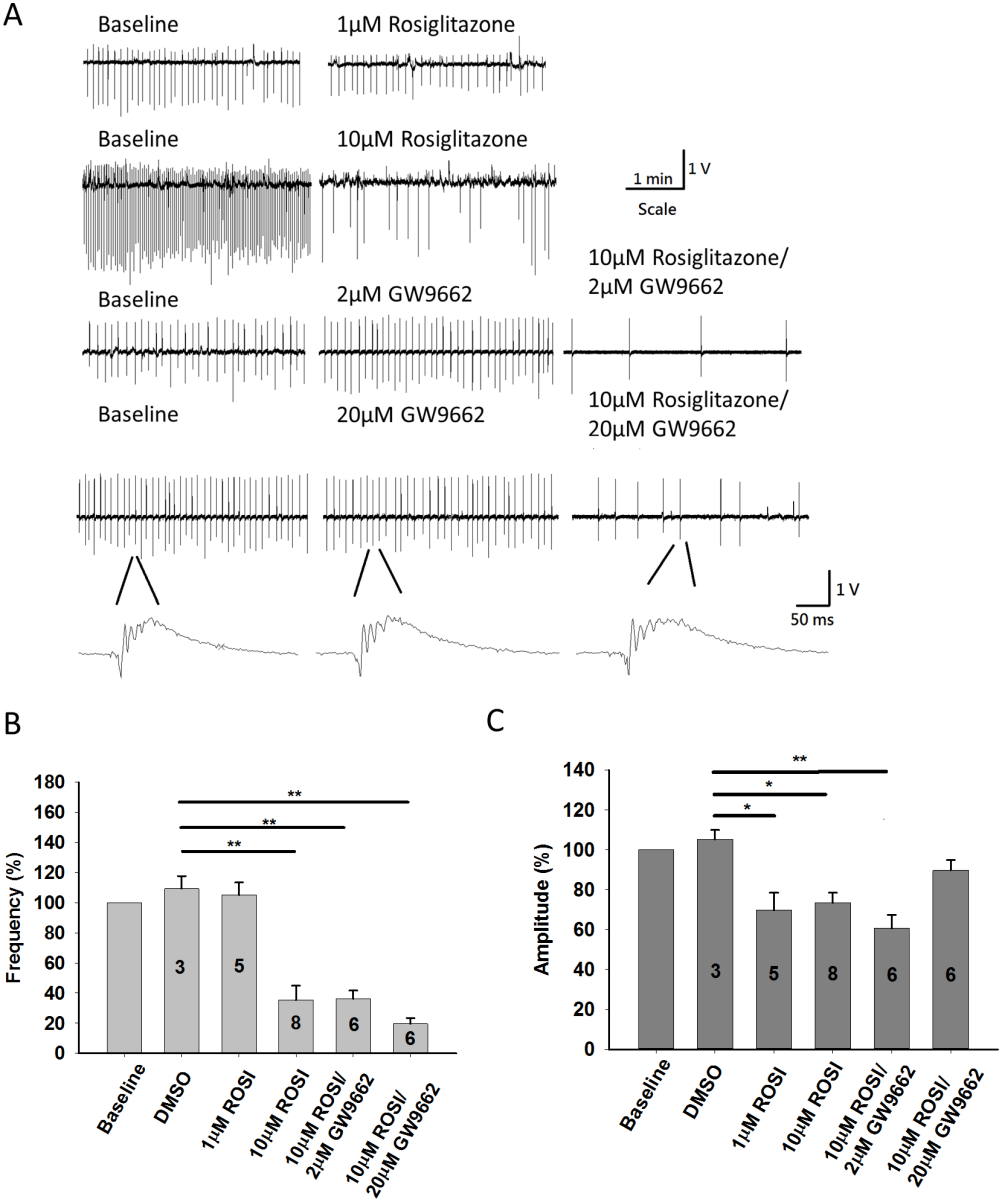
Epileptiform discharges induced by Mg^2+^-free artificial cerebral spinal fluid (ACSF). (A) Field-potential recording from the CA1 region of acute hippocampal slices showing regular spontaneous activity induced by Mg^2+^ free ACSF. Application of 10μM rosiglitazone significantly decreased firing frequency of epileptiform discharges. Pretreatement with 2μM or 20μM GW9662 did not block the anti-convulsive effect of 10μM rosiglitazone. (B) Quantification of spike frequency before and after application of 1μM and 10μM rosiglitazone showing that10μM rosiglitazone can significantly suppresses spike frequency. Application of 2μM or 20μM GW9662 didn’t block the attenuation of spike frequency from 10μM rosiglitazone. (C) Quantification of spike amplitude during control ACSF, GW9662, and rosiglitazone infusion. Application of 1μM and 10μM can suppress spike amplitude. The decrease in spike amplitude was reversed by pretreatment with 20μM GW9662. *P <0.05 **P<0.01.

As previously reported, Mg^2+^-free ACSF can induce ictal-like and interictal-like epileptiform activities [7, 8, 21]. Because we discarded the entorhinal cortex, ictal-like events frequently ceased after several bursts and we were able to only take slices that showed regular interictal-like epileptiform activities for experiments. Interictal spontaneous epileptiform discharges were provoked successfully in thirty-one hippocampal slices from sixteen different animals. Three slices developed ictal-like events and were discarded for analysis. The amplitudes and frequencies of the spontaneous events induced by Mg^2+^ free ACSF were counted and measured manually. After a stable recording for 15 mins, we normalized the spike frequencies and amplitudes recorded in next 5 mins as baseline. Spikes recorded 25–30 mins after rosiglitazone/GW9662 application were normalized with baseline. One way ANOVA and post-hoc analysis with LSD are used to compare different experimental groups. Baseline interictal spike frequency was 0.21 ± 0.02 Hz and the amplitude was 1.32 ± 0.17 V (n = 28). Experiments with DMSO, rosiglitazone or GW9662 infusion were significantly different in interictal spike frequency (F = 17.08, *P*<0.001) and spike amplitude (F = 3.814, *P* = 0.016). As shown in Fig 1B and 1C, application of 20μL DMSO made no changes on spike amplitude (105.29 ± 4.63% compared with baseline, n = 3, *P* = 0.42) and spike frequency (109.38 ± 8.27% compared with baseline, n = 3, *P* = 0.40). Application of 1μM rosiglitazone suppressed spike amplitude to 69.79 ± 8.66% compared with baseline (n = 5), but the frequency of interictal spikes was not changed (105.16 ± 8.31% compared with baseline, n = 5). By post-hoc analysis with LSD method, 1μM rosiglitazone suppressed spike amplitude (*P* = 0.032) but not spike frequency (*P* = 0.61) in comparison with DMSO group. Application of 10μM rosiglitazone suppressed spike amplitude to 73.49 ± 5.03% compared with baseline (n = 8) and frequency to 35.34 ± 9.49% compared with baseline (n = 8). Comparing with application of 1μM rosiglitazone by one way ANOVA with LSD post-hoc analysis, application of 10μM rosiglitazone significantly suppressed interictal spike frequencies (*P*<0.001) but not spike amplitude (*P* = 0.891).

Next, we explored the role of PPARγ activation in the anti-convulsive effect from 10μM rosiglitazone by administering 2μM and 20μM GW9662 to block PPARγ activation10 minutes before and during rosiglitazone application. Application of 2μM GW9662 had no effect on spike frequency (104.34 ± 3.48% compared with baseline, n = 6, *P* = 0.40) and amplitude (91.72 ± 9.37% compared with baseline, n = 6, *P* = 0.47). Application of 10μM rosiglitazone combined with 2μM GW9662 suppressed spike frequency to 36.04 ± 5.69% compared with baseline (n = 6, *P*<0.001, Fig 1B), while decreasing spike amplitude to 60.68 ± 6.73% compared with baseline (n = 6, *P* = 0.019, Fig 1C). Finally, we tested 20μM GW9662 before and during administration of 10μM rosiglitazone and found no effect of 20 μM GW9662 on spike frequency (93.45 ± 3.78% compared with baseline, n = 6, *P* = 0.32) or amplitude (103.32 ± 1.89% compared with baseline, n = 6, *P* = 0.20). However, spike frequency was still significantly suppressed following 10μM rosiglitazone application (19.61 ± 3.80% compared with baseline, n = 6, *P*<0.001, Fig 1B) indicating GW9662 does not affect rosiglitazone’s effect on suppression of spontaneous spikes induced by Mg^2+^ free medium. Interestingly, pretreatment with 20μM GW9662 block the effect of 10μM rosiglitazone on suppressing spike amplitude (89.72 ± 5.61% compared with baseline, n = 6, *P* = 0.24, Fig 1C). By post-hoc analysis with LSD, treatment with 10μM rosiglitazone/20 μM GW9662 significantly suppressed spike frequency (*P* = 0.010) but not spike amplitude (*P* = 0.240) in comparison with DMSO group. These results suggest rosiglitazone modulates spike frequencies via non-PPARγ pathway and modulates spike amplitudes via PPARγ activation.

### Effect of rosiglitazone 1μM/10μM on Schaffer collateral—CA1 synaptic transmission

**Fig 2:**
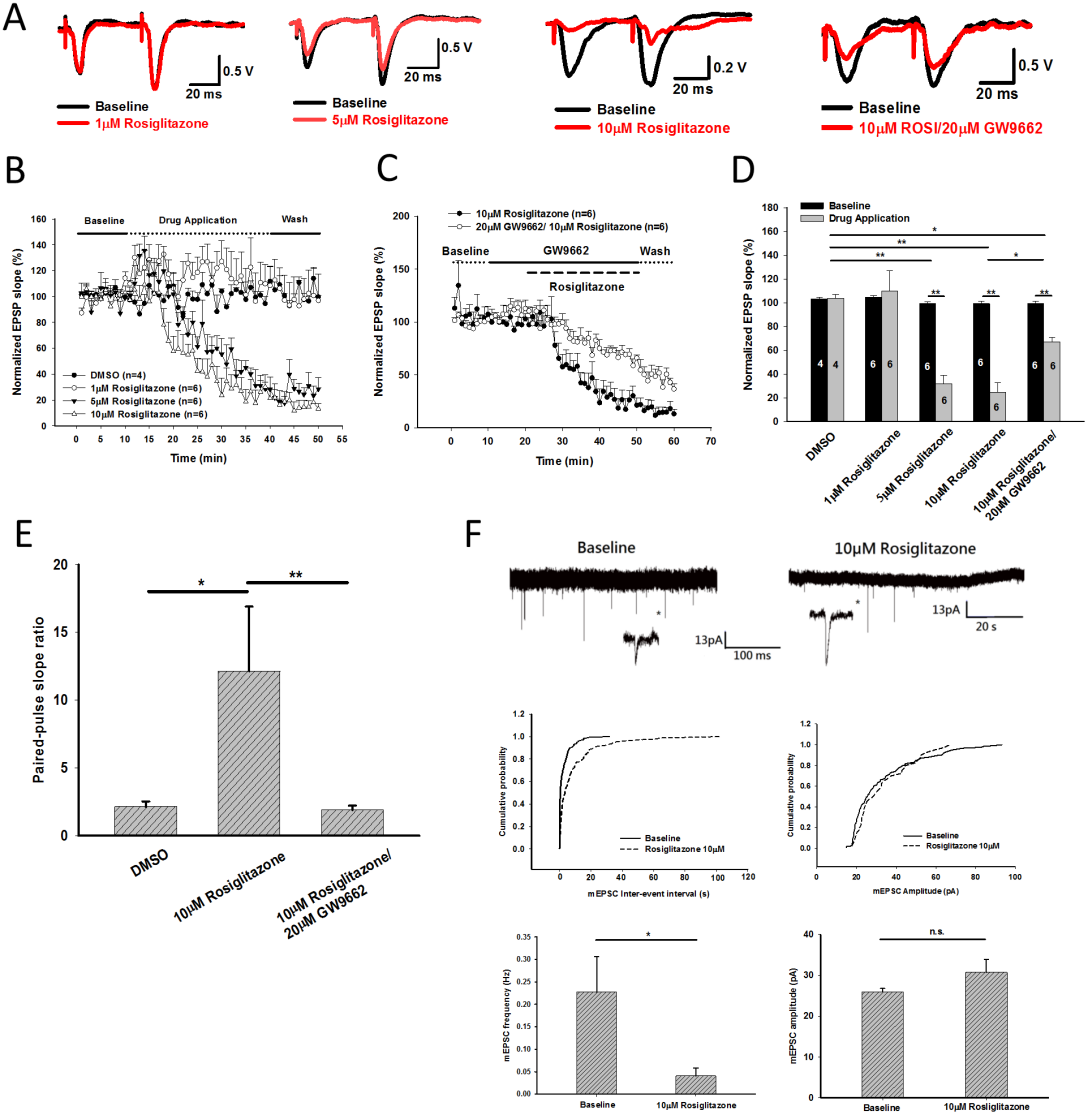
Effects of rosiglitazone on CA1-Schaffer collateral field evoked potentials (fEPSPs) and CA1 pyramidal cell miniature excitatory postsynaptic currents (mEPSC). (A) Representative traces of fEPSPs before (black color) and after application of 1,5,10μM rosiglitazone with/without 20μM GW9662 treatment for 30 minutes (red color). (B) The slope of first fEPSPs were suppressed significantly by 5 and 10μM rosiglitazone. (C) Cotreatment with 20μM GW9662 partially reversed the suppression of fEPSP slope induced by 10μM rosiglitazone. (D) Quantification of fEPSP slope after rosiglitazone/GW9662 treatment for 30 minutes. (E) Application of 10μM rosilglitazone significantly increased pair-pulse ratio on CA1-Schaffer collateral fEPSPs, which indicates rosiglitazone significantly suppresses presynaptic neurotransmitter release. This effect can be completely reverse by pretreatment with 20μM GW9662. (F) Miniature EPSCs recorded on CA1 pyrimidal cells. Application of 10μM rosiglitazone significantly suppressed mEPSC frequency but not amplitude. Cumulative probability of mEPSC inter-event interval (P<0.001 by Kolmogorov–Smirnov test) and mEPSC amplitude (P = 0.18 by K-S test) from the representative trace of mEPSC were illustrated. *P<0.05 **P<0.01.

In the next step, we induced field evoked-postsynaptic potentials (fEPSP) in Schaffer collaterals as shown above by administering paired electrical stimulation (interval 50 ms) once per minute. Baseline fEPSPs were recorded for 30 minutes, to establish baseline values, then ligands were added to the ACSF and fEPSPs were recorded for next 30 minutes. The final five fEPSP slopes taken during baseline and drug application were used for quantification. D-(-)-2-Amino-5-phosphonopentanoic acid (APV) 50μM was added in ACSF during the whole recording. As shown in fig 2, application of 1μM rosiglitazone had no effect on EPSP slope (109.74 ± 17.14% compared with baseline, Fig 2B and 2D). Application of 5μM and 10μM rosiglitazone significantly suppressed first EPSP but not the second one on our paired pulse protocol (Fig 2A). Application of 5μM/10μM rosiglitazone significantly suppressed first fEPSP slope to 31.79 ± 7.04% (n = 6, P<0.001) and 24.37 ± 8.38% (n = 6, *P*<0.001) compared with baseline, respectively (Fig 2B). Then we tested the role of PPARγ pathway. Pretreatment with 20μM GW9662 (PPARγ antagonist) before application of 10μM rosiglitazone still suppressed fEPSP slope to 67.05 ± 3.60% (n = 6, p<0.001) compared with baseline. This decrement was significantly less than treatment with 10μM rosiglitazone only (*P* = 0.01 with repeat-measured ANOVA, Fig 2C and 2D).

The fEPSP slope ratio from paired pulse stimulation in 50ms interval after DMSO and 10μM rosiglitazone treatment were 2.14 ± 0.39 and 12.14 ± 4.75, respectively (*P* = 0.014) (Fig 2E). This finding indicated rosiglitazone may inhibit presynaptic neurotransmitter release significantly. Paired-pulse slope ratio was reduced to 2.14 ± 0.39 when we pretreated 20μM GW9662 before rosiglitazone application (*P* = 0.005) (Fig 2E), indicating the presynaptic effect of rosiglitazone was mediated from PPARγ activation. Thus, we recorded miniature excitatory-postsynaptic currents (mEPSCs) on hippocampal CA1 pyramidal cells. Application of 10μM rosiglitazone significantly suppress mEPSC frequency from 0.23 ± 0.08 Hz to 0.04 ± 0.02 Hz (P = 0.049) but not mEPSC amplitude (*P* = 0.147) (Fig 2F). We also presented one example trace to calculate cumulative probability of mEPSC inter-event interval (*P* < 0.001 by K-S test) and amplitudes (*P* = 0.18 by K-S test) in Fig 2F. This finding indicated rosiglitazone suppressed presynaptic neurotransmitter release but not post-synaptic neurotransmitter binding efficiency.

### Neuroprotection of rosiglitazone against excitotoxicity in hippocampal slice cultures

**Fig 3:**
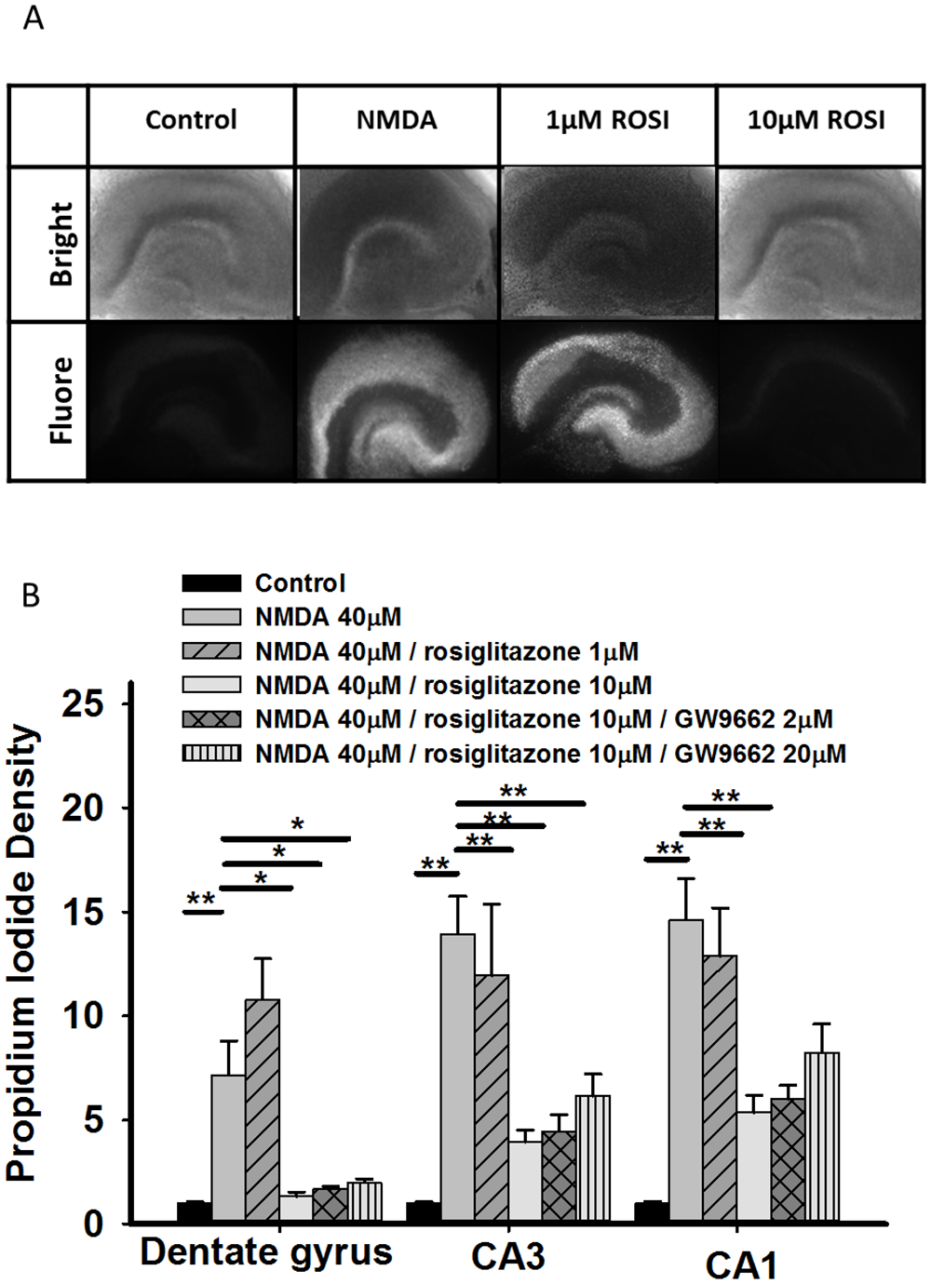
Rosiglitazone protected cultured hippocampal slices from (n-Methyl-D-Aspartate) NMDA-induced excitotoxicity. (A) Representative images of fluorescence intensity after NMDA treatment with/without rosiglitazone rescue. Bright: bright-field microscopy, Fluore: fluorescence microscopy (B) Quantitative results for fluorescence intensity after NMDA treatment with/without rosiglitazone rescue. The PI fluorescence intensity was normalized by those slices treated in control medium. Application of 40μM NMDA significantly increased PI density in dentate gyrus, CA3 and CA1 areas of hippocampus. Application of 10μM rosiglitazone significantly suppressed neuronal damage by NMDA in the dentate gyrus, CA3 and CA1 areas of the hippocampus. Pretreatment with 20μM GW9662 partially reverse the neuroprotective effect of 10μM rosiglitazone in CA1 neurons. **P <0.01 compared with PI intensity of NMDA treated slices.

The effect of rosiglitazone on excitotoxicity induced by NMDA was explored using PI density quantification in the dentate gyrus, CA3, and CA1. We added 40μM NMDA to induce neuronal injury in hippocampal slice cultures. In experimental group, rosiglitazone was applied on the hippocampal slices, 30 minutes before the application of NMDA. After 1 hour we removed the culture medium containing NMDA and added back rosiglitazone, then continued the treatment for 24 hours. PI density quantification was done 24 hours after NMDA application. For the group treated with GW9662 (the PPARγ antagonist), GW9662 was applied 30 minutes before rosiglitazone. These experiments were repeated 3 times, each time containing 10–18 slices in the control group and 4–13 slices in treatment groups. PI density quantification and normalization were performed independently in each experiment. We found that NMDA treatment increased PI density in dentate gyrus, CA3 and CA1 of hippocampus to 7.2±1.6, 13.9 ± 1.8, 14.6 ± 2.0, respectively (Fig 3B). Application of 1μM rosiglitazone had no significant effect compared with slices treated with NMDA. Co-Application of 10μM rosiglitazone and NMDA significantly suppressed PI density in the dentate gyrus, CA3 and CA1 to 1.3 ± 0.2 (*P* = 0.005), 3.9 ± 0.6, *P* <0.001) and 5.4 ± 0.8 (*P* = 0.001) compared with NMDA treated slices. Pretreating with 2μM GW9662 did not antagonize the protective effect of 10μM rosiglitazone on PI density in the dentate gyrus, CA3 and CA1 (Fig 3B). PI density in CA1 after treatment with 20μM GW9662/10μM rosiglitazone revealed no significant difference comparing with NMDA treatment (*P* = 0.145, Fig 3B). These findings suggest that 10μM rosiglitazone significantly suppresses NMDA induced excitotoxicity in cultured hippocampal slices, and GW9662 in high dose partially antagonized the effect of rosiglitazone. This indicated the neuroprotection from rosiglitazone is partially mediated from activation of the PPARγ pathway.

## Discussion

In this study, we found that rosiglitazone can suppress epileptiform discharges induced by Mg^2+^-free medium in CA1 pyramidal cells. Rosiglitazone’s effect on discharge frequency cannot be blocked by the PPARγ antagonist GW9662, but its effect on discharge amplitude can be reversed by GW9662. Also, using CA1-Schaffer collateral field recording, we determined that rosiglitazone significantly suppressed fEPSP and increased pair-pulse slope ratio, which indicating rosiglitazone inhibit presynaptic neurotransmitter release. We also recorded mEPSC on hippocampal CA1 pyramidal cells. Rosiglitazone inhibit mEPSC frequency significantly but not mEPSC amplitude. In hippocampal slice culture, rosiglitazone significantly attenuated NMDA-induced excitotoxicity, which is partially mediated from activation of PPARγ pathway. It indicated that rosiglitazone suppressed NMDA receptor-mediated epileptiform discharges by non-PPARγ mediated mechanisms. Rosiglitazone suppressed synaptic transmission by inhibiting presynaptic neurotransmitter release and protected hippocampal slice from NMDA excitotoxicity partially by activation of PPARγ pathway.

The interictal epileptiform discharges of hippocampal CA3 region induced by Mg^2+^ free medium are generated by the presence of recurrent excitatory connections among neighboring pyramidal cells and can transmit to CA1 and dentate gyrus. These interictal discharges resemble the paroxysmal depolarizing shifts (PDS), which has been considered for a long time the hallmark of focal epileptiform interictal activity[19]. Traub et al. have suggested that synchronized after-hyperpolarization is generated by NMDA receptors, which provides a prolonged depolarization of the dendrites of neuron cells, resulting in dendritic voltage-gated Ca^2+^ bursting, which then drive the repetitive bursts of action potentials at the soma[22]. Thus, NMDA receptors are the key factors of low extracellular magnesium-induced epileptiform activity in hippocampal slices[8, 10, 23]. In our study, rosiglitazone successfully suppressed this low extracellular magnesium-induced epileptiform activity in hippocampal slices, a method commonly used for screening chemicals with anti-convulsive effects[21, 24]. Excessive glutamate may lead to neuronal over-excitation and seizures as shown in animal studies, in which microinfusions of the glutamate metabolizing enzyme (glutamine synthase) inhibitor into hippocampus induced recurrent seizures, loss of hippocampal neurons, and resulted in pathological changes comparable to hippocampal sclerosis[25]. In human studies, increases inactivity of the glutamine synthetase or increased expression of NMDA receptor 1 transcript in hippocampal neurons have been reported in patients with TLE[26, 27]. We found that rosiglitazone can suppress NMDA receptor-mediated epilepiform discharges in vitro. NMDA-induced excitotoxity of cultured hippocampal slices was also attenuated by co-treatment with10μM rosiglitazone. This suggests that clinically, rosiglitazone may have potential to treat patients with temporal lobe epilepsy.

Interestingly, the effect of rosiglitazone on reducing discharge frequency cannot be blocked by GW9662, a PPARγ antagonist, but the effect on reducing discharge amplitude can be blocked by inhibition of PPARγ activation. These findings suggest that the frequency and amplitude of spontaneous discharges on hippocampal neurons induced by Mg^2+^ free medium are adjusted by different mechanisms. The initiation of those spontaneous discharges is NMDA-dependent. The dendritic calcium spike occurs during the secondary burst of those discharges[22]. Rosiglitazone can reduce voltage-gated Ca^2+^ channel (VGCC)-mediated Ca^2+^ current in cultured hippocampal neurons. This phenomenon can be blocked by pretreating another PPARγ antagonist T0070907[18]. Taken these together, rosiglitazone can reduce the frequency of NMDA-mediated spontaneous discharges in hippocampal neurons by non-PPARγ pathway. In the other hand, rosiglitazone reduced amplitude of spontaneous discharges by a PPARγ dependent pathway, possibly by inhibition of VGCC Ca^2+^ current. Rosiglitazone may suppress inflammation by several PPARγ-independent pathways, such as Janus kinase (JAK) and the STAT signaling pathways [28, 29]. JAK/STAT pathway was ever reported to play an essential role in the induction of NMDA receptor-dependent long-term depression [30]. Further studies are needed to clarify the role of JAK/STAT pathway for those NMDA receptor-mediated discharges.

Because rosiglitazone significantly suppressed NMDA receptor-mediated spontaneous activity in hippocampal CA1 neurons, we tested rosiglitazone on synaptic transmission of CA1-Schaffer collateral pathway. We found rosiglitazone primarily worked on inhibition of presynaptic neurotransmitter release, which is PPARγ dependent. Alteration of presynaptic release machinery in structure and function were found in mice after pilocarpine-induced status epilepticus[31]. This phenomenon could contribute to the development of chronic epileptic state. Rosiglitazone significantly suppressed presynaptic vesicle release and made it a possible rescue to this neuronal damage induced by status epilepticus. Recently, Nenov et al. found rosiglitazone can rescue hyperactivity of dentate gyrus granular cells by a presynaptic mechanism. They concluded that through PPARγ activation, rosiglitazone enhanced hippocampal cognitive function from regulation of presynaptic vesicular proteins critical for proper glutamatergic neurotransmitter release (SNARE-associated proteins), synaptic transmission, and short-term plasticity in Tg2576 APP mice, a model of Alzheimer’s disease[32]. VGCC opening is also a major trigger for spontaneous glutamate release at hippocampal synapses[33]. Possibly, through inhibition of VGCC, rosiglitazone could further inhibit presynaptic glutamate release. The presynaptic effect of rosiglitazone made it a potential cognitive enhancer and also an anti-convulsant.

PPARs are ligand-activated transcription factors that belong to the nuclear hormone receptor family that play an important role in glucose and lipid metabolism, as well as cell proliferation and differentiation. PPARγ expression in CNS is limited to the basal ganglia, dentate gyrus of hippocampus, thalamus, brainstem, and astrocytes[14]. There is evidence that PPARγ agonists could improve the neurological outcomes in the variable central nervous system diseases, such as Alzheimer’s disease[34], multiple sclerosis[35], Parkinson’s disease[36], and acute cerebral ischemia[37]. Furthermore, PPARγ agonists have been shown to suppress inflammation following ischemic and hemorrhagic stroke [37, 38]. PPARγ also regulates the expression of some important antioxidative enzymes such as catalase, SOD1, and GST that ameliorate oxidative stress [39]. Both proinflammatory mediators and oxidative stress induce neurotoxicity by activated microglia [40] and PPARγ agonists may exert neuroprotection against CNS diseases by inhibition of these microglia-mediated process. PPARγ activation has been previously found to be beneficial to epileptic neuronal injury as rosiglitazone reduced hippocampal neuronal loss in lithium-pilocarpine induced status epilepticus in rats [15, 16]. The mechanisms for this neuroprotection include attenuation of inflammatory responses and inhibition of oxidative stress and in both these studies, the PPARγ antagonist T0070907 blocked these effects. In PTZ-induced seizures in mice, the anticonvulsant effect of acute pioglitazone was occluded by GW9662 [41]. Our study supply more solid evidence that rosiglitazone treated seizures by inhibiting presynaptic glutamate release, which has not been reported before. This made a new window to look for effect of PPARγ pathway in CNS.

Rosiglitazone was released by GlaxoSmithKline in 1999 for diabetes patients as an insulin sensitizer. However, a meta-analysis in the New England Journal of Medicine in 2007 concluded that rosiglitazone was associated with a significant increase in the risk of myocardial infarction [42] and the drug was suspended from the European market. In the US and Canada, the drug can still be prescribed, but with serious warnings and precautions that it should be used only when all other oral antidiabetic agents fail to give adequate glycemic control or are inappropriate due to contraindications or intolerance. As a result, although there are unexpected side effects that limit the use of TZDs, many studies focusing on physiologic and therapeutic relevance of PPARγ are still going on [43].

In our study, rosiglitazone suppressed amplitude of NMDA receptor-mediated epileptiform discharges, inhibited presynaptic neurotransmitter release and recued NMDA excitotoxicity via PPARγ activation. Rosiglitazone suppressed frequencies of NMDA receptor-mediated epileptiform discharges via non-PPARγ pathway. We suggest that rosiglitazone may be a potential agent to treat patients with TLE.
